# Rapamycin‐nanoliposomes target the mTORC1‐mediated autophagy–lysosomal and NLRP3/Caspase‐1 pathways to inhibit nucleus pulposus cell senescence in intervertebral discs

**DOI:** 10.1002/btm2.70165

**Published:** 2026-08-03

**Authors:** Hui Xing, Miao Yu, Jiabin Liu, Runzhi Zhao, Xuezheng Ai, Rong Tang, Tong Zhu, Yuqing Li, Lu Jiang, Quanfang Wei, Youying Huang, Yu Guo, Tao Jiang, Bo Huang

**Affiliations:** ^1^ Department of orthopaedics Xinqiao Hospital Chongqing China; ^2^ Clinical Medical Research Center Xinqiao Hospital Chongqing China; ^3^ Biomedical Analysis and Testing Center, Basic Medical College Army Medical University Chongqing China; ^4^ Department of Radiology Daping Hospital Chongqing China

**Keywords:** autophagy‐lysosome, cell senescence, intervertebral disc degeneration, mTORC1, NLRP3/Caspase‐1, P27, rapamycin‐nanoliposomes

## Abstract

Nucleus pulposus (NP) cell quiescence maintains intervertebral disc homeostasis, while mTORC1 regulates autophagy–lysosomal function and inflammatory secretion to preserve quiescence—rapamycin specifically targets mTORC1. Herein, we fabricated rapamycin‐nanoliposomes (rapa‐lipos) via ultrasonic dispersion, thin‐film dispersion, and filtration to improve rapamycin bioavailability, investigating their role in inhibiting the senescence phenotype of NP cells through *β*‐gal staining, lysosomal staining, transmission electron microscopy, ELISA, and cell cycle inhibitors. The mechanistic effects of rapa‐lipos on mTORC1, NLRP3/Caspase‐1 pathway (NCP) and autophagy–lysosomal pathway (ALP) were also analyzed by western blotting, immunofluorescence (IF), Si‐RNA (raptor), and PCR. In vivo, rapa‐lipos were injected into rat intervertebral disc with IL‐1*β*‐induced degeneration, assessed via HE staining, x‐ray, MRI, and IF. Rapa‐lipos exhibited high encapsulation efficiency, favorable drug loading, uniform particle size, and controlled release, suppressing NP cell senescence‐related phenotypes (morphological changes, elevated IL‐1β/TNF‐*α* secretion, increased *β*‐gal activity, lysosomal dysfunction, upregulated P21/P16 and reduced P27 expression). Mechanistically, rapa‐lipos targeted‐inhibited mTORC1, then blocked NCP and activated ALP to maintain NP cell quiescence. In vivo, x‐ray, MRI and histological evaluation confirmed rapa‐lipos mitigated intervertebral disc degeneration. Collectively, rapa‐lipos target mTORC1‐mediated NCP and ALP to inhibit NP cell senescence, offering a promising strategy for intervertebral disc degeneration prevention.

**Translational Impact Statement**

We developed rapamycin nanoliposomes via a facile preparation method with good biophysical properties, to target mTORC1 and modulate autophagy‐lysosomal and NLRP3/Caspase‐1 pathways. This inhibits nucleus pulposus cell senescence and mitigates disc degeneration in rats, offering a translational therapeutic strategy for degenerative intervertebral disc disease.

**Abbreviations**
ALP: autophagy–lysosomal pathway
EE: encapsulation efficiency
HE: hematoxylin–eosin
IF: immunofluorescence
NCP: NLRP3/Caspase‐1 pathway
NP: nucleus pulposus
rapa‐lipos: rapamycin‐loaded nanoliposomes
SASP: senescence‐associated secretory phenotype
SD rats: Sprague‐Dawley rats
TEM: transmission electron microscopy
WB: western bolt
*β*‐gal: *β*‐galactosidase

## 1 BACKGROUND

The intervertebral disc is a closed microenvironment composed of fibrous annulus and nucleus pulposus, which contains many NP cells.^1^ Owing to the lack of nutrient supply to the disc, NP cells are in a quiescent state.^2^ Research on intervertebral disc aging has revealed that the NP cells are typically in the quiescent (G0) phase of the cell cycle, which is the key to preventing cell senescence and disc degeneration.^3^, ^4^ Cell quiescence not only refers to the non‐proliferation of the cell cycle, but to its feature of cell‐free senescence.^4^ Chronic inflammation can also accelerate the aging process in individuals.^5^ Compared with TNF‐*α*, IL‐6 and IL‐17, IL‐*β* is the most potent proinflammatory factor, which is secreted by senescent cells and can cause normal cell senescence and intervertebral disc degeneration.^6^ Rapamycin inhibits the production of inflammatory factors, maintains cell quiescence, and slows the aging process.^7^

Senescence is a state of irreversible cell cycle arrest associated with aging,^8^, ^9^ and increased mTORC1 phosphorylation drives cell senescence, which is characterized by growth arrest, increased senescence marker *β*‐gal, autophagy–lysosome dysfunction, and increased secretion of proinflammatory factors.^10^, ^11^ Intervertebral disc degeneration results in a large amount of inflammation, which is regulated by mTORC1.^12^ mTORC1 is a central regulator and determines whether cells exiting the cell cycle enter quiescence or senescence.^12^ Rapamycin‐mediated modulation of mTORC1 inhibits proinflammatory production via the NCP.^13^ Moreover, the inhibition of mTORC1 enhances the function of ALP, promotes the phagocytosis of harmful molecules inside cells, and inhibits cell senescence and the aging of tissues.^10^

However, rapamycin is insoluble in water and has a short half‐life in vivo, which limits its use.^14^ Liposomes encapsulate hydrophobic drugs, which can improve their water solubility, control their release, and enhance long‐term biological effects.^15^ Compared to other nanoparticles, nano‐liposomes have excellent properties, such as biocompatibility, drug loading capacity, controlled release, and biodegradability.^16^, ^17^, ^18^ In addition, different from displaced rapamycin, rapamycin‐nanoliposomes (rapa‐lipos) can target lysosomes of senescent cells and achieve lysosomal function activation at a lower drug dose.^19^ To enhance the biological effects of rapamycin, we constructed rapa‐lipos, which have high drug loading capacity, high encapsulation efficiency, and the ability to controlled release of rapamycin.^20^

We hypothesize that rapa‐lipos regulate the ALP and NCP by targeting mTORC1, thereby inhibiting inflammation production and enhancing lysosomal function. This leads to the NP cells being in the quiescent stage and delays cell senescence and intervertebral disc degeneration. Therefore, the research focus of this paper is as follows: ① rapa‐lipos are constructed, and their particle size, drug loading rate, encapsulation rate, and sustained release of rapamycin are evaluated. ② In vitro experiments reveal that rapa‐lipos protect against the senescence of NP cells by targeting mTORC1 and then mediating the ALP and NCP. ③ In vivo, the effects of rapa‐lipos are shown to delay intervertebral disc degeneration.

## 2 METHODS

### 2.1 Nanoliposome construction and performance testing

#### 2.1.1 Construction of the rapa‐lipos

Reagents: rapamycin (GlpBio, Cat. No. GC15031); trehalose (MACKLIN, D807342‐5 g); 1,2‐dipalmitoyl‐sn‐glycero3‐phosphocholine (DPPC, MACKLIN, D863688‐100 mg); tert‐Butanol (MACKLIN, T819476).

Using ultrasonic dispersion, thin film dispersion and filter methods,^21^, ^22^ rapamycin was dissolved in tert‐butanol, trehalose was dissolved in water, and 1,2‐dipalmitoyl‐sn‐glycero3‐phosphocholine was dissolved in tert‐butanol. A total of 1.0 mg of rapamycin, 5.0 mg of DPPC and 10 mg of trehalose were mixed, placed in a 60°C water bath for 30 min for evaporation, and then lyophilized in a lyophilizer (Telstar, Vacuum freezing:Lyoquest‐85) for 3 h to obtain liposomes. One milliliter of ultrapure water was added to the liposomes, which were then processed with 200 W of a multiprobe ultrasonic pulverizer (KS‐650SDN) for 30 min (ultrasonicated for 20 s, ultrasonicated for 5 s, and placed in an ice bath; the ultrasound treatment parameters, in accordance with the usage guidelines of the ultrasound equipment and the research methods of previous scholars.^22^) To obtain a liposome suspension with a uniform particle size, rapa‐lipos were hydrated and filtered through a filter (0.45 μm) (Betime‐FF365), after which they were irradiated at 18 Gy for sterilization. Blank liposomes without rapamycin were used as a control as described above.

#### 2.1.2 Calculation of the rapa‐lipo drug loading capacity, encapsulation efficiency

Rapa content in particles: To determine the rapa content in the rapa‐lipos, the above rapa‐lipo(rapamycin content = 1 mg/mL) was mixed dissolved in 4 mL of ultrapure water, filtered for 2 mL (rapamycin content = 0.5 mg, total weight of particles = rapamycin + DPPC + trehalose = 8 mg) and placed in a 3500 D dialysis bag (Solarbio, YA1080) clamped shut and put into a 500 mL beaker filled with 500 mL of deionized water, with continuous magnetic stirring at 25°C, and the deionized water in the beaker was replaced every 3 h. Twenty‐four hours later, the liquid in the dialysis bag was collected. Fifty microliters of this mixture was dissolved in 1 mL of deionized water to construct the test solution. Fifty microliters of test solution was taken according to the instructions of the rapamycin ELISA Kit (FANKEW, F9021‐A) to determine the amount of rapa in the liposomes. The absorbance was finally measured with an enzyme labeling instrument (SpectraMAX M2) at 450 nm. Finally, according to the ELISA kit's guidelines, the absorbance and rapamycin standard curves are obtained, and the “Rapa content in particles” is calculated.

Total content of rapamycin: the above rapa‐lipo dissolve 10ul in 1 mL of deionized water to construct the test solution. Follow the rapamycin ELISA Kit (FANKEW, F9021‐A) guidelines to take 50ul of test solution. Absorbance was measured on a SpectraMAX M2 (450 nm wavelength). The actual rapa‐lipos concentration was determined according to absorbance and standard curve.

$$\text{Rapa loading}\left(\%\right)=\begin{array}{l} \left(\text{Rapa content in particles}/\text{total weight of particles}\right)\\ \times 100\%; \end{array}$$

$$\text{Rapa encapsulation rate}\left(\%\right)=(\text{Rapa content in particles}/\text{Total content of rapamycin})\times 100\%\left[33\right].$$

#### 2.1.3 Sustained‐release rate of assay

The above liposomes (rapamycin content: 0.5 g) were dissolved in 0.5 mL of ultrapure water, filtered and placed in a 3500D dialysis bag (Solarbio, YA1080) clamped shut, 2 mL of PBS was added externally, and 2 mL of the filtered liquid was collected at 3, 24 h, 4, 7, 14, 21, and 28 days. The mixture was subsequently replenished to the same volume as before, and the collected mixture was frozen in a −30°C refrigerator. The concentration of rapa in the mixture was measured using a rapamycin ELISA Kit (FANKEW, F9021‐A), and the ELISA procedure was performed according to the manufacturer's protocol.

#### 2.1.4 Determination of particle size and surface potential

Thirty microlitres of liposomes (1 mg/mL rapa content) were suspended in 1 mL of water, filtered and vortexed thoroughly, and analyzed by a Malvern nanoscale particle sizer (Zetasizer Nano, Nano ZS), in which the temperature was set to 25°C, the measurement angle was 90°, and the detection wavelength was 633 nm.

#### 2.1.5 TEM observation

Negative staining: Thirty microliters of liposomes (1 mg/mL rapa content) were suspended in one milliliter of water, filtered and vortexed well, equally spread on a copper mesh; dyed with 15 μL 1% phosphotungstic Acid (PTA, pH 7.0) and stained for 1 min, and allowed to evaporate at room temperature. Finally, the copper mesh was observed under a TEM (JEM‐1400plus, JEOL) (indicated magnification: 150 kx).

### 2.2 NP cell extraction from medullary tissue and senescence phenotype analysis

#### 2.2.1 NP cell extraction and culture

SD rats (BEIJING HFK BIOSCIENCE CO., Ltd., 8 weeks old, 180–220 g, male) were sacrificed by intraperitoneal injection of a high dose of sodium pentobarbital (>150 mg/kg) in compliance with previously reported procedures,^4^ and the tails were meticulously extracted under aseptic conditions. NP cells obtained from their tail intervertebral discs show no senescence characteristics, which is a good model for studying cell quiescence and is also easy to obtain. To liberate the NP cells, the gel‐like medullary tissue in the central intervertebral disc was removed and processed for 1 h at 37°C using 0.2% type II collagenase (Solarbio, C8150). Following filtering, the NP cells were moved to DMEM/F12 (Gibco, C11330500BT), which was enhanced with 1% penicillin–streptomycin (HyClone, SV30010) and 10% fetal bovine serum (Gibco, 10099‐141). Every 3 days, fresh culture medium was added to the NP cells, which were being cultivated at 37°C with 5% CO_2_. Third‐generation cells were used in all of the cell tests. The cell experiments were grouped as follows: ① G0: 1% FBS + DMEM/F12 + 1% penicillin–streptomycin; ② IL‐ 1*β*: 1% FBS + DMEM/F12 + 1%penicillin–streptomycin + IL‐1*β* (10 ng/mL) (perprotech, AF‐211‐11B‐10UG); ③ rapa‐lipos: 1% FBS + DMEM/F12 + 1% penicillin–streptomycin + IL‐1*β* (10 ng/mL) + rapa‐lipos (rapamycin, 50 μg/mL. This is the recommended concentration as stated in the instruction manual; DPPC, 250 μg/mL); and ④ blank: 1% FBS + DMEM/F12 + 1% penicillin–streptomycin + blank liposome (DPPC, 250 μg/mL). All NP cells treated for 7 days and replaced with fresh medium every 2 days. The gross morphology was observed under a light microscope (Olympus, IX73).

#### 2.2.2 Β‐Gal staining

Following the *β*‐gal staining method,^23^, ^24^ after grouping and treatment, the NP cells had been fixed with the fixative contained in the senescent *β*‐gal staining kit (Beyotime, C0602) for 15 min after being washed with PBS, aspirated, and incubated in *β*‐gal staining solution at 37°C for the entire night. The cells were then analyzed under a light microscope for blue staining, which is indicative of senescence. The percentage of cells that were favorably stained was calculated.

#### 2.2.3 Lysosomal tracker red staining

The LysoTracker Red working mixture was prepared according to previous methods^25^: First, 1 μL of LysoTracker Red (Beyotime, C1046) was added to 15 mL of culture medium (1% FBS + DMEM/F12 + 1% penicillin–streptomycin) and incubated at 37°C in an incubator with 5% CO_2_ and 0.2 mM ascorbic acid. After grouping and treatment, NP cells were removed from the original medium; the working mixture was added. The mixture was incubated with the cells at 37°C for 30 min. The staining working mixture solution was removed, fresh cell culture medium was added, and the lysosomal staining intensity was observed via fluorescence microscopy (Olympus, IX73). Semiquantitative analysis was performed with ImageJ.

#### 2.2.4 TEM observation of lysosomal autophagy

As previously described^26^, NP cells after grouping and treatment were centrifuged and fixed in 2.5% glutaraldehyde for 24 h. The cells were cleaned, dehydrated with gradient acetone, then embedded after being fixed for 1 h in 1% osmium tetroxide. A Leica EM UC7 ultramicrotome was used to create ultrathin 90‐nm‐thick slices, which were then stained with dioxin acetate and lead solution and analyzed using a TCM (JEM‐1400plus, JEOL) (indicated magnification: “G0,” “IL‐1*β*,” “rapa‐lipos” for 30kx; “blank” for 20kx). A Gatan US1000 high‐resolution digital camera was used to shoot the pictures.

#### 2.2.5 ELISA detection of inflammatory secretion

T75 culture flasks were used to cultivate NP cells. After grouping and treatment, the cells were rinsed five times with PBS, 10 mL of DMEM/F12 medium was added, and the medium was then cultivated for 12 h. For the detection of IL‐1*β*, the medium was collected and thoroughly lyophilized; 200 μL of DMEM/F12 was added. IL‐1*β* was measured according to the instructions of the rat IL‐1*β* ELISA kit (FANKEW, F2923‐A). The absorbance was then measured at 450 nm using an enzyme labeling device (Molecular Devices, Spectra Max M2), with DMEM/F12 serving as a control. The 10 mL medium was tested immediately (not lyophilized) for TNF‐*α* detection in accordance with the guidelines provided by the mouse TNF‐*α* ELISA Kit (invitrogen, BMS607‐3).

### 2.3 Low‐serum methods to induce NP cell quiescence (EdU, and cell cycle PI)

#### 2.3.1 EdU staining

NP cell proliferation was assessed via the BeyoClick‐TM EdU Cell Proliferation Kit with Alexa Fluor555 (Beyotime, C0075S) to detect EdU DNA incorporation.^27^ The related experimental groups were used according to step 2.1, and the medium was fully changed every 2 days. Control group: P3 NP cells were cultured with complete medium (10% FBS + DMEM/F12 + 1% penicillin–streptomycin) for 48 h. Briefly, ① the cells were incubated in 6‐well plates for 7 days and then washed 3 times with PBS. One milliliter of DMEM/F12 and 1 mL of EdU working solution (20 μM) were added, and the mixture was incubated for 2 h. ② The medium was removed, and 1 mL of fixative (from the kit) was added for 15 min at room temperature. ③ The fixative was removed, and the cells were washed three times with washing solution and removed. ④ The cells were washed with 1 mL of permeabilizing solution at room temperature for 15 min and then removed. ⑤ The cells were washed twice with washing solution and removed. ⑥ Fifty‐five microliters of Click Additive Solution was added to each well, and the mixture was incubated for 30 min at room temperature in the dark. ⑦ Step ⑤ was repeated. ⑧ The nuclei of the cells were stained with Hoechst 33342 and then removed as described in ⑤. The cells were observed and photographed under a fluorescence microscope (Olympus, IX73), and the number of EdU‐positive cells was semiquantitatively determined via ImageJ.

#### 2.3.2 PI staining

In accordance with an earlier cell cycle methods,^3^ the NP cells after grouping and treatment were collected, trypsinized, and given twice PBS washes. After that, single‐cell suspensions were fixed in 70% ethanol at −20°C. After centrifugation and several PBS washes, the fixed cells were resuspended in PBS. Next, 500 μL of PI staining solution (BD PharmingenTM, 550825) was added to the resuspended cells, which were allowed to lie at room temperature for 20 min in the dark. After that, the cells were placed on ice, and a flow cytometer (Beckman Coulter, GALL IOS) was used to collect at least 10,000 event cells. ModFit LT 4.1 was used for examining each sample.

### 2.4 Analysis of the effects of rapa‐lipos on the ALP and NCP

#### 2.4.1 IF staining

NP cells were grouped, treated, and cultured on cell culture dishes (NEST, 801001), fixed in 4% paraformaldehyde (BOSTER, AR068) for 15 min, and penetrated with 0.3% Triton X‐100 (Biofrox, 1139ML100) for 15 min at room temperature. Anti‐NLRP3 (ABclonal, A5652, 1:200) was used to incubate the cells overnight at 4°C following a 30‐min blocking period with 3% BSA. The following secondary antibodies were then added to the cells and incubated for 1 h at 37°C without light: CY3, goat anti‐rabbit IgG (Abbkine, A22220), or goat anti‐rabbit IgG (H + L) Alexa Fluor 647 (Bioworld, BS10033). Lastly, DAPI (Beyotime, P0131) was used to counterstain the nuclei, and confocal microscopy (Zeiss, LSM880) was used to acquire fluorescence pictures. ImageJ was used to perform a semiquantitative analysis of the fluorescence intensity.

#### 2.4.2 WB

After grouping and treatment, NP cell protein extracts were obtained using lysis buffer (Beyotime, P0013) that contained a protease inhibitor (Beyotime, P1005) and a phosphatase inhibitor cocktail II (GLPBIO, GK10012). A BCA protein assay (Beyotime, P0012S) was used to measure the total protein content. Protein extracts underwent future PAGE (ACE, ET15012Lgel, 12% concentration: for IL‐1RI, P62; ACE, ET15420Lgel, 4%–20% concentration: for LC3B, P16, P21, P27, Caspase‐1; ACE, ET15412Lgel, 4%–12% concentration for P‐mTOR). After being separated, the proteins were put onto a 0.22 μm PVDF membrane (Merck Millipore, ISEQ00010) for immunoblotting (Overall, it takes 2 h, but LC3B, P16, P21, and P27 takes 1 h and P‐mTOR takes 2.5 h). Then the membrane was blocked with 5% skim milk (Servicebio, GC310001‐100 g) for 1 h and washed with TBST for 30 min. The membrane was incubated with primary antibodies against P62 (ABclonal, A19700, 1:500), LC3B (CST, 3868T, 1:1000), P‐mTOR (HUABIO, HA600094, 1:1000), Caspase‐1(pro) & Caspase‐1(cleavad) (ABclonal, A0964SP, 1:5000), IL‐1RI (SAB, 41670, 1:500), P16 (Abclonal, Cat. No. A23882, 1:1000), P21 (Abclonal, Cat. No. A19094, 1:1000) and P27 (HUABIO, Cat. No. ET1608‐61, 1:2000) and *β*‐actin (ABclonal, AC006, 1:1000) for an entire night. The membrane was washed with TBST for 30 min and then the HRP‐conjugated goat anti‐rabbit IgG (H + L) secondary antibodies (ABclonal, AS014, 1:10000) was used to incubate the membranes for 1 h at room temperature. The membranes were washed with TBST for 30 min and then detected using an enhanced chemiluminescence (ECL) Super Kit (ABclonal, RM02867). In order to expose the protein bands, the membranes were lastly subjected to ChemiDoc Imaging System (Bio‐rad).

*Special attention*: For PVDF membranes of P‐mTOR after exposure, the Stripping Buffer (CWBIO, CW0056M) is required for elution antibody. The process of blocking with 5% skimmed milk and incubating the primary and secondary antibodies was carried out again (For the primary antibodies: incubating mTOR (CST, 2983 T, 1:1000) with P‐mTOR membrane). Finally, ECL was added to expose the membrane. ImageJ was used to quantify the results.

#### 2.4.3 Real‐time quantitative qPCR

NP cells were grouped and treated, the total cellular RNA was extracted via the TRIzol method (RNAiso Plus, TAKaRa, 9109). Total RNA (1 μg) was reverse transcribed via ABScript Neo RT Master Mix for qPCR with gDNA Remover (ABclonal, RK20433). PCR was performed on a real‐time PCR system (Roche, Cobas z480) using 2 × Universal SYBR Green Fast qPCR Mix (RK21203). PCR analysis was performed via the following protocol: denaturation at 95°C for 3 min, followed by 45 cycles of 95°C for 5 s and 60°C for 34 s. The levels of target genes were analyzed via the 2^−ΔΔCt^ method, and GAPDH was used as an internal reference. The primer sequences are listed in Table 1.

**TABLE 1:** The relevant synthetic primer sequences are listed below.

Primer name	Sequence (5′ to 3′)
P62_F	CCTAGAGGGGCTTTGGTT
P62_R	TTTCTGGTGCAGGTGTTG
Il1b_F	GAGAGCATCCAGCTTCAAA
Il1b_R	TCATCATCCCACGAGTCA
Il1rl1_F	ACTCACCGTTACCTTCCT
Il1rl1_R	GTTAATCGCACCTCCTCTT
LC3_F	TACAGCACTCCAGCAATG
LC3_R	AACACAGATTCCAACTTCCT
GAPDH_F	GACATGCCGCCTGGAGAAAC
GAPDH_R	AGCCCAGGATGCCCTTTAGT

#### 2.4.4 Preparation and transfection of raptor‐siRNAs

To further confirm the ability of the rapa‐lipos to target mTORC1, we silenced mTORC1 via siRNA technology to validate the role of the ALP and NCP in the effects of the rapa‐lipos. We tested the ability of three sets of siRNAs (Sangon Biotech) to silence raptor (the core component of mTORC1) and selected the siRNA with the strongest silencing effect for the following experiments. The target sequences were as follows: siRNA Raptor (sense, 5′ to 3′): CCU UCA UUC UUG CUG UAA UTT. siRNA Raptor (antisense, 5′ to 3′): AUU ACA GCA AGA AUG AAG GTT. In accordance with the manufacturer's instructions, the NP cells were recultured and transfected with PEI Max 40K reagent (Polysciences, 24765), and the detailed procedures for the transfection protocols followed the manufacturer's recommendations. The final concentration of the siRNAs was 10 nM (SiRNA:PEI Max 40K = 1:3). Twenty‐four hours after transfection, the transfection medium was replaced with fresh growth medium, and different intergroup treatments were performed. IF and WB were used to detect changes in the ALP and NCP levels after siRNA‐mediated silencing of raptor.

The raptor‐siRNA groups were as follows: ① G0: 1% FBS + DMEM/F12 + 1% penicillin–streptomycin; ② IL‐ 1*β*: 1% FBS + DMEM/F12 + 1% penicillin–streptomycin + IL‐1*β* (10 ng/mL) (perprotech, AF‐211‐11B‐10UG); ③ Si‐RNA(raptor): 1% FBS + DMEM/F12 + 1% penicillin–streptomycin + IL‐1*β* (10 ng/mL) + Si‐RNA(raptor); ④ Negative control Si‐RNA: 1% FBS + DMEM/F12 + 1% penicillin–streptomycin + negative control‐siRNA. All raptor‐siRNA groups were treated for 7 days and replaced with fresh medium every 2 days.

### 2.5 Evaluation of the effect of Rapa‐lipos on cell cycle arrest

Rapamycin exerts a cell cycle‐arresting effect to maintain cellular quiescence, thereby inducing the upregulated expression of the cell cycle inhibitors P16, P21, and P27 to varying degrees.^28^ Thus, we evaluated the impact of rapa‐lipos on cellular quiescence by analyzing the expression levels of these inhibitors. Total proteins were extracted from cells in each group after treatment, with the detailed procedure performed in accordance with the “WB” section. The primary antibodies used were as follows: P16 (Abclonal, Cat. No. A23882, 1:1000), P21 (Abclonal, Cat. No. A19094, 1:1000), and P27 (HUABIO, Cat. No. ET1608‐61, 1:2000).

### 2.6 Rapa‐lipos protect against caudal intervertebral disc degeneration in Sprague‐Dawley (SD) rats

#### 2.6.1 X‐rays and MRI were assessed for disc degeneration

SD rats (BEIJING HFK BIOSCIENCE CO., LTD, 6 weeks old, 150–180 g, male) were selected and anesthetized with 2% (mass/volume ratio) sodium pentobarbital (40 mg/kg). First, a microsyringe needle was slowly inserted into the caudal intervertebral disc of each rat at a depth of 0.2 cm and then withdrawn immediately after injection. (The height of the intervertebral disc before the operation was normal. It is approximately equal to half of the sum of the heights of the previous and next intervertebral discs.) The animal experimental groups were as follows: ① Blank‐lipos group: 2 μL of blank liposomes were added; ② IL‐1*β* group: 2 μL of IL‐1*β* (10 ng/mL) was added; ③ IL‐1*β* + rapa‐lipos group: 2 μL of IL‐1*β* (10 ng/mL) + 2 μL of rapa‐lipos (rapamycin, 50 μg/mL) were added; and ④ untreated group: no injection. One month later, the rats were intraperitoneally injected with a high dose of pentobarbital sodium (>150 mg/kg), and the caudal tissue was removed for x‐ray imaging (randomly, *n* = 4) and MRI (randomly, *n* = 3). X‐rays were acquired via a small animal imaging system (BRUKER, Xtreme II). For MRI, T2‐weighted cross‐sectional images were obtained by BRUKER BioSpec 70/20USR, and the slice thickness was set to 0.8 mm.

#### 2.6.2 Histological evaluation of the caudal intervertebral discs(HE and IF)

The rat caudal intercalated discs were then fixed, decalcified, paraffin‐embedded, and sectioned at a thickness of 10 μm on a microtome (Leica, RM2255). For HE staining: All the sections were sequentially immersed in xylene solvent, graded‐alcohol solution, and finally sections were stained with HE solution. The sections were photographed for the general morphology under a light microscope (Olympus, IX73).

For IF staining: For details, refer to the above experimental procedure “IF staining.” The simple process is as follows: All the sections (*n* = 3, randomly) were sequentially fixed in 4% paraformaldehyde, penetrated with 0.3% Triton X‐100 and blocking period with 3% BSA. Primary antibody: anti‐aggrecan (ABclonal, A12045, 1:200), overnight at 4°C. The secondary antibody: goat anti‐rabbit IgG (H + L) Alexa Fluor 647 (Bioworld, BS10033), 1 h at 37°C without light. Lastly, DAPI was used to counterstain the nuclei. The sections were photographed using a light microscope (Olympus, IX73) and ImageJ was performed for the fluorescence intensity.

### 2.7 Statistical analysis

Comparisons were made via analysis of variance (ANOVA) via SPSS 27.0 software (SPSS). The data are expressed as the means ± standard deviations. Comparisons between groups were performed via *t*‐tests or one‐way ANOVA; differences were considered statistically significant at *p*< 0.05.

## 3 RESULTS

### 3.1 Rapa‐lipo characterization

To promote the ability of rapamycin to target mTORC1, we first aimed to construct rapa‐lipos to improve the excellent physicochemical properties of rapamycin, such as its water solubility, high encapsulation rate, high drug loading rate, sustained release, nanoscale size, and electric charge.

After hydration and ultrasonic treatment, the solid rapa‐lipos (Figure 1a‐1) changed into a milky and translucent gel‐like structure(Figure 1a‐2), which was nanoscale in size according to the TEM results (Figure 1b). A particle size analyzer was used to analyze the particle diameter, which ranged from 141 to 165 nm (Figure 1c) and showed a positive charge (Figure 1d).

**FIGURE 1:**
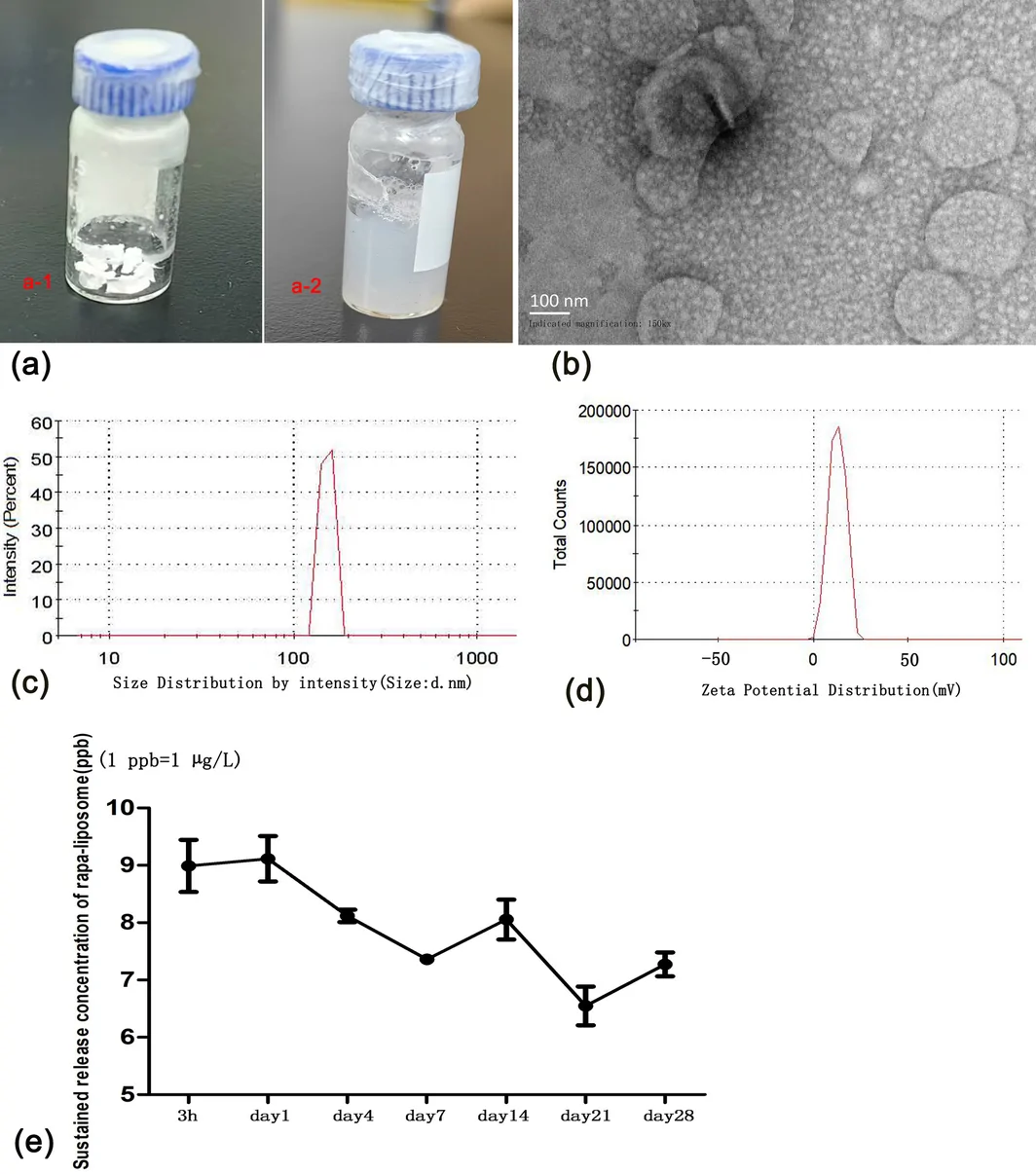
The physical and chemical properties of rapa‐lipos. (a) Rapa‐lipo appearance and morphology (a‐1): After lyophilization, liposomes showed solid state; (a‐2): The state of rapa‐lipos at the end of the preparation); (b) micromorphology of the rapa‐lipos observed via transmission electron microscopy (TEM); (c) and (d) particle size and charge of the rapa‐lipos (temperature: 25°C, measurement angle: 90°, detection wavelength: 633 nm); (e) sustained‐release rate performance of the rapa‐lipos over 28 days (*n* = 3, mean ± SD).

The encapsulation rate of rapamycin in the liposomes was 80.3% ± 3.99%, and the drug loading rate was 5.02% ± 0.25% (Table 2). In vitro, the rapa‐lipos exhibited sustained release of rapamycin for 28 days, and the concentration of released rapamycin reached a maximum on the first day at 9.116 ± 0.396 ppb and decreased progressively with increasing time. The concentration of rapamycin at 28 days was 7.273 ± 0.208 ppb (Figure 1e) (1 ppb = 1 μg/L). The appearance, microscopic size, charge, and sustained‐release properties of the rapa‐lipos were consistent with the concept of nanoliposomes. Therefore, these rapa‐lipos can be used as tools for studying the biological and pharmacological properties of rapamycin.

**TABLE 2:** The drug loading rate and encapsulation rate of rapa‐lipos.

Category	Data1	Data2	Data3	Mean ± Standard Deviations
Drug loading(%)	4.94	4.82	5.30	5.02 ± 0.25
Encapsulation rate(%)	79.08	77.16	84.83	80.36 ± 3.99

### 3.2 Rapa‐lipos inhibited the senescent phenotype of NP cells (gross‐morphology, SASP secretion, increased *β*‐gal, and lysosomal changes)

Senescent cells often exhibit changes in cell morphology, increased secretion of SASP (inflammatory factors, such as IL‐1*β* and TNF‐*α*), and increased *β*‐gal and lysosomal dysfunction.^10^, ^11^ This chapter focuses on how rapa‐lipos change the phenotype of senescent NP cells.

Gross morphology: Under a light microscope, G0 NP cells were observed to be round and pike shaped after treatment with low serum, with no significant change in morphology after treatment with the rapa‐lipo or blank formulation. After treatment with the “IL‐1*β*” formulation, the volume of the NP cells increased, and the cells became flattened, no longer being round or pike‐shaped (Figure 2a).

**FIGURE 2:**
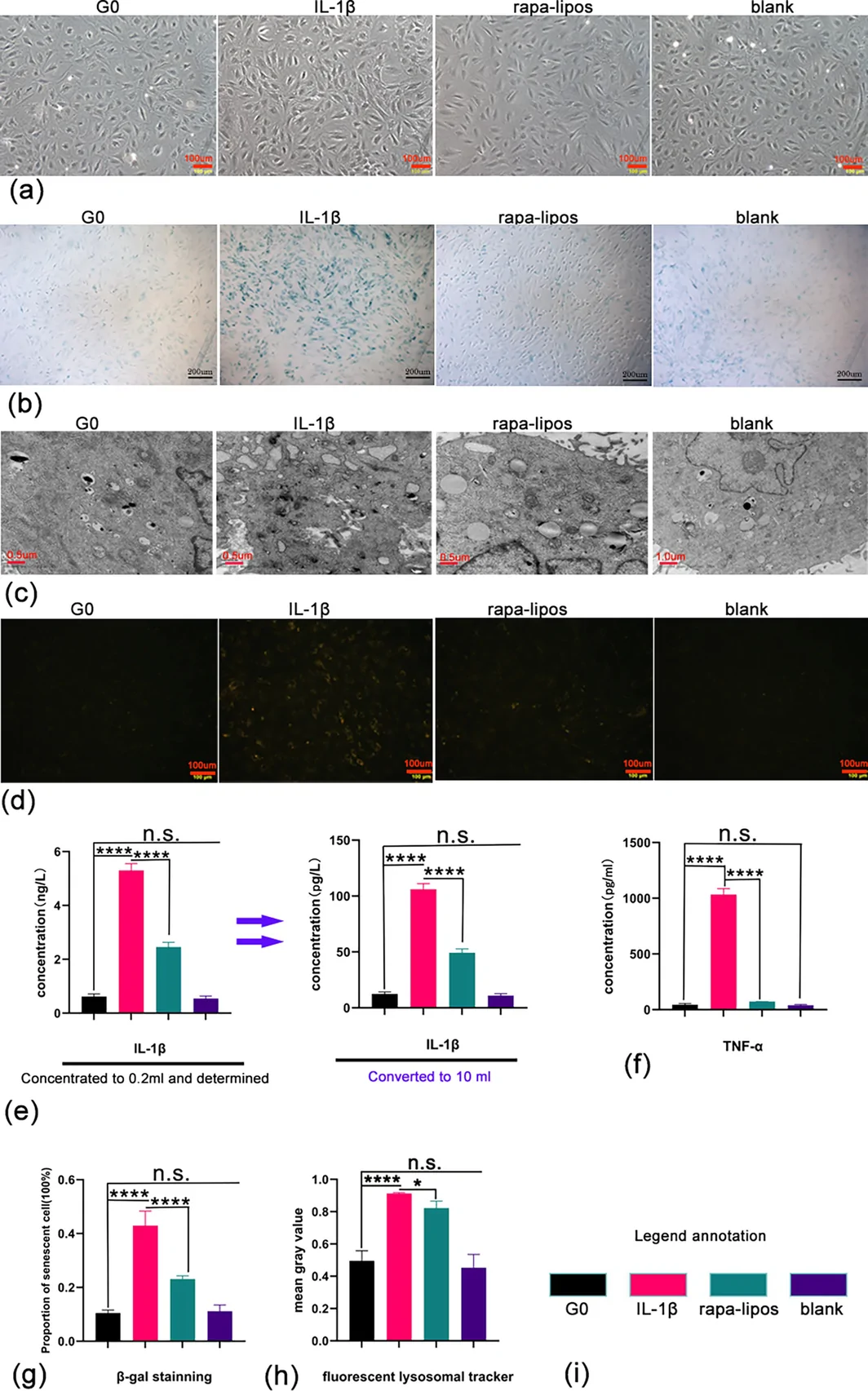
Characterization of senescent NP cells and analysis of the antiaging effect of rapa‐lipos. Gross morphology of NP cells under a bright‐field microscope (a), Gross morphology of *β*‐gal‐stained NP cells (b), TEM observation of lysosomal morphology of NP cells (c), Fluorescence imaging of NP cells with a fluorescent lysosomal tracke (d), ELISA for IL‐1*β* (e) and TNF‐*α* (f) secretion (*n* = 3, mean ± SD, one‐way ANOVA, *p*< 0.05 indicates a statistically significant difference, ****p< 0.0001); the percentage of senescent cells after *β*‐gal staining (g) (*n* = 4, mean ± SD, one‐way ANOVA, *p*< 0.05 indicates a statistically significant difference, ****p< 0.0001, n.s. indicates no significance); The mean gray value analysis of fluorescent lysosomal tracker (h) (*n* = 4, mean ± SD, one‐way ANOVA, *p*< 0.05 indicates a statistically significant difference, *p< 0.05, ****p< 0.0001, n.s. indicates no significance); the legend annotation (i).

ELISA: Compared with the “G0” group of NP cells, the IL‐1*β*‐treated group of NP cells secreted more IL‐1*β* (Figure 2i) (Figure 2e, 12.38 ± 1.84 vs. 105.95 ± 5.09, *p*< 0.0001; *n* = 3) and TNF‐*α* (Figure 2i) (Figure 2f, 44.20 ± 10.94 vs. 1032.46 ± 53.78, *p*< 0.0001; *n* = 3). Compared with that in the “IL‐1β” group, the secretion of IL‐1*β* (Figure 2i) (Figure 2e, 105.95 ± 5.09 vs. 49.14 ± 3.52, *p*< 0.0001; *n* = 3) and TNF‐*α* (Figure 2i) (Figure 2f, 1032.46 ± 53.78 vs. 72.24 ± 0.30, *p*< 0.0001; *n* = 3) was lower in the “rapa‐lipos” group. Moreover, IL‐1*β* and TNF‐*α* secretion was not significantly different between the “Blank” and “G0” groups of NP cells (Figure 2i) (Figure 2e,f, *p* >0.05; *n* = 3).

*β‐gal staining*: As NP cells senescent, they are stained dark blue by *β*‐gal, and rapa‐lipos has shown an ability to block this change (Figure 2b). Compared with that in the “G0” group of NP cells, the number of senescent NP cells in the “IL‐1*β*” group increased (Figure 2i) (Figure 2g, 0.43 ± 0.05 vs. 0.10 ± 0.01, *p*< 0.0001, *n* = 4), whereas compared with that in the “IL‐1β” group, the number of senescent cells in the “rapa‐lipos” group significantly decreased. (Figure 2i) (Figure 2g, 0.23 ± 0.01 vs. 0.43 ± 0.05, *p*< 0.0001, *n* = 4). The “blank” group had no effect on NP cell senescence (Figure 2i) (Figure 2g, 0.11 ± 0.02 vs. 0.10 ± 0.01, *p*< 0.0001; *n* = 4).

*Lysosome observation*: TEM revealed the integrity of lysosome morphology (round organelles with black spots inside) inside the NP cells in the “G0” and “blank” groups (Figure 2c). IL‐1*β* induced senescent cells showed the destruction of lysosomal morphology, whereas rapa‐lipos could maintain the integrity of lysosomes in an inflammatory environment (Figure 2c).

Additionally, regarding NP cells with a red fluorescent lysosomal tracker, the results showed that the fluorescence intensity was weaker in the “G0” and “blank” groups (Figure 2d), which did not differ from each other (Figure 2i) (Figure 2h, 0.50 ± 0.06 vs. 0.45 ± 0.06, *p* >0.05, *n* = 4). Compared with that of the G0 group, the fluorescence intensity of the NP cells was greater after IL‐1*β* treatment (Figure 2d) (Figure 2i) (Figure 2h, 0.915 ± 0.01 vs. 0.50 ± 0.06, *p*< 0.0001, *n* = 4), but compared with the “IL‐1*β*” group, the “rapa‐lipos” treatment significantly reduced the fluorescence intensity (Figure 2d) (Figure 2i) (Figure 2h, 0.82 ± 0.04 vs. 0.915 ± 0.01, *p*< 0.05, *n* = 4).

### 3.3 NP cells simulate cell quiescence under low‐serum conditions

NP cells are located in the intervertebral disc and lack a nutrient supply, resulting in the NP cells being in the quiescent phase and maintaining the normal function of the intervertebral disc.^3^, ^4^ This is the reason why NP cells were cultured in vitro with low serum in this study.

EdU staining: EdU staining was performed to verify the quiescence of NP cells. The “G0,” “IL‐1*β*,” “rapa‐lipos,” and “blank” groups (Figure 3b) did not exhibit significant cell proliferation. There was no significant difference in the cell proliferation rate among the groups (Figure 3c) (Figure 3d, *p* >0.05, *n* = 4). P3 NP cells were cultured in complete medium for 48 h (Figure 3b), and the proliferation rate was significantly greater than that of the other groups (Figure 3c) (Figure 3d, *p*< 0.0001, *n* = 4).

**FIGURE 3:**
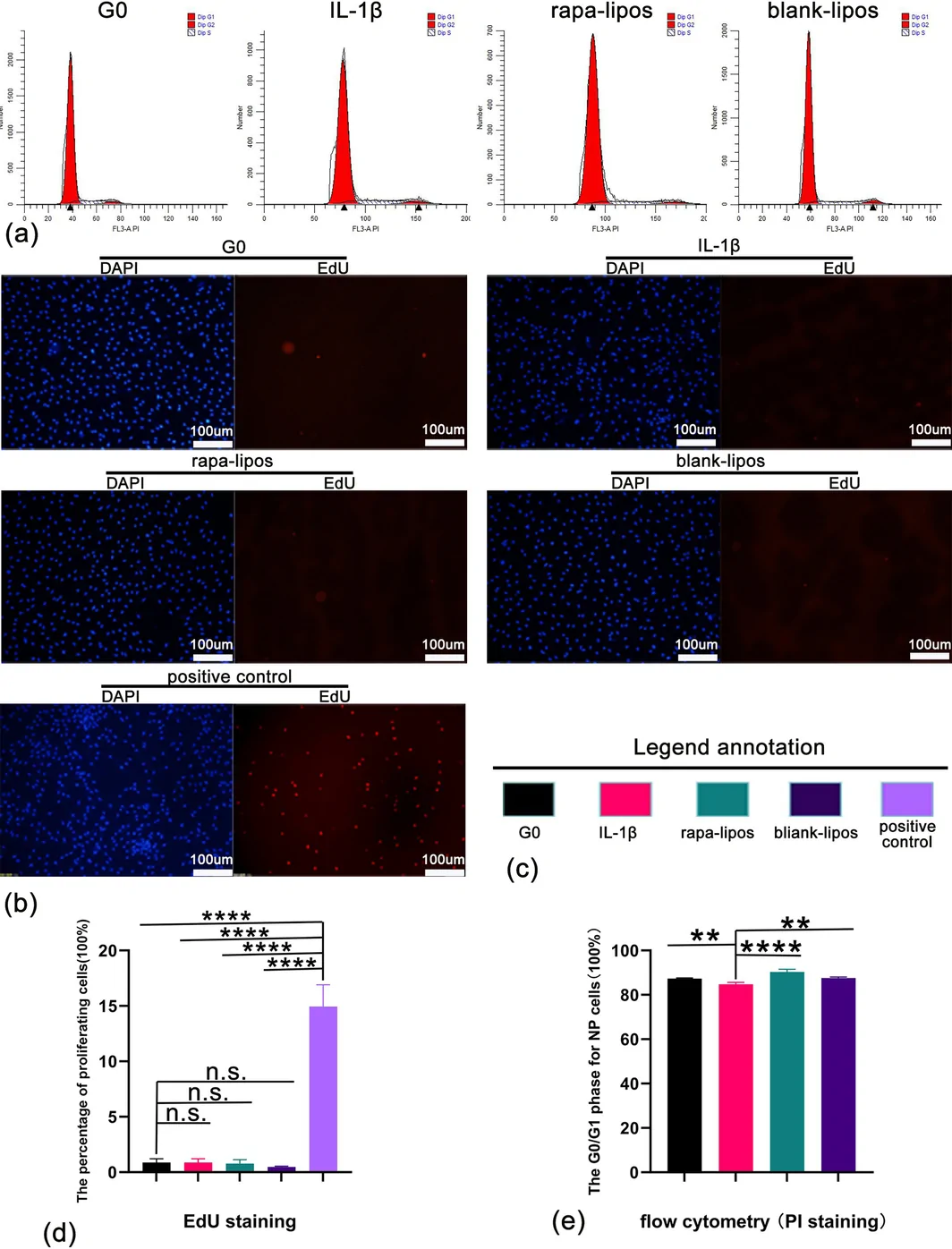
Observation of quiescent NP cells. The cell cycle was detected via flow cytometry PI staining (a) and EdU staining (b). The legend annotation (c). The proportion of proliferating NP cells according to EdU staining (d) (*n* = 4, mean ± SD, one‐way ANOVA, *p*< 0.05 indicates a statistically significant difference, ****p< 0.0001, n.s. indicates no significance). The flow cytometry analysis of the percentage of NP cells in the G0/G1 stage (e) (*n* = 3, mean ± SD, one‐way ANOVA, *p*< 0.05 indicates a statistically significant difference, *p< 0.05, **p< 0.01, n.s. indicates no significance).

*PI‐flow cytometry*: PI staining and flow cytometry analysis revealed that the vast majority of the NP cells in each group were in the G0/G1 phase(Figure 3a). The percentages of NP cells in the G0/G1 phase were as follows: “G0” (Figure 3c) (Figure 3e, 87.26 ± 0.36, *n* = 3), “IL‐1*β*” (Figure 3c) (Figure 3e, 84.71 ± 0.95, *n* = 3), “rapa‐lipo” (Figure 3c) (Figure 3e, 90.30 ± 0.1, *n* = 3), and “blank” (Figure 3c) (Figure 3e, 87.55 ± 0.46, *n* = 3).

Low‐serum was used to simulate the microenvironment of low nutrient supply within the intervertebral disc. Using EdU staining and PI‐flow cytometry, we found that almost all NP cells did not proliferate after IL‐1*β*‐induced or IL‐1*β* + rapa‐lipo cotreatment, indicating that the low‐serum culture model can successfully simulate the quiescent state of NP cells. Cell growth arrest was observed in both quiescent and senescent NP cells.

### 3.4 Inhibition of NP cell senescence by rapa‐lipos targeting mTORC1: The NCP and ALP (PCR, WB, IF)

The WB results revealed that, compared with that in the “G0” group, the membrane receptor IL‐1R expression in the “IL‐1*β*” group increased(Figure 4a) (Figure 4d) (Figure 4e, 1.10 ± 0.32 vs. 0.51 ± 0.05, *p*< 0.01; *n* = 3). There was no statistically significant difference in IL‐1R expression between the “IL‐1*β*” group and the “lipo‐rapos” group (Figure 4a) (Figure 4d) (Figure 4e, 1.10 ± 0.32 vs. 1.05 ± 0.21, *p* > 0.05, *n* = 3).

**FIGURE 4:**
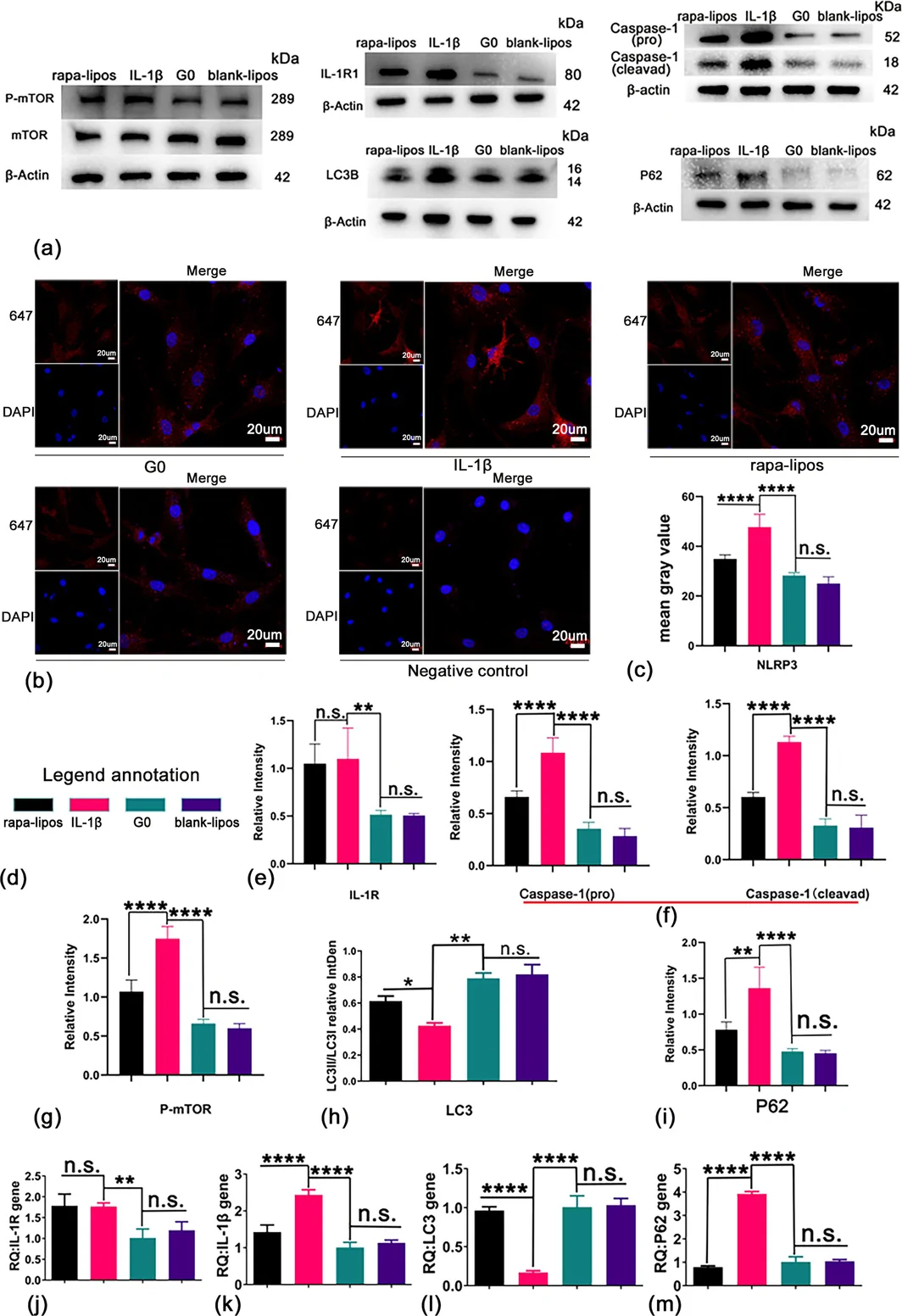
Molecular mechanism of rapa‐lipos targeting of mTORC1 mediates the NCP and ALP to maintain NP cell quiescence. NP cells were subjected to WB (a) and IF (b) to analyze the expression of P‐mTOR (g); the NCP‐related proteins IL‐1R (e), NLRP3 (c), and Caspase‐1 (pro) and Caspase‐1 (cleavad) (f); and the ALP‐related proteins LC3 (h) and P62 (i). PCR was performed to analyze the mRNA expression of the NCP‐related genes IL‐1R (j) and IL‐1*β* (k) and the ALP‐related genes LC3 (l) and P62 (m). (WB and PCR: *N* = 3, IF: *N* = 4; mean ± SD, one‐way ANOVA, *p*< 0.05 indicates a statistically significant difference, *p< 0.05, **p< 0.01, ****p< 0.0001, n.s. indicates no significance.) The legend annotation (d).

Elevated levels of mTORC1 phosphorylation indicate impaired intracellular autophagolysosomal function and increased intracellular inflammatory cytokine synthesis.^12^, ^29^ Moreover, mTORC1 phosphorylation positively reflects the expression level of P‐mTOR protein. Therefore, the level of P‐mTOR protein expression represents a change in autophagolysosomal function and the synthesis and secretion of inflammatory factors (such as IL‐1*β*, TNF‐*α*).

After G0 NP cells were stimulated with IL‐1*β*, P‐mTOR expression (P‐mTOR/mTOR) was increased (Figure 4a) (Figure 4d) (Figure 4g, 1.75 ± 0.16 vs. 0.66 ± 0.05, *p*< 0.0001; *n* = 3). Additionally, the expression of the NCP‐related proteins NLRP3 (Figure 4b) (Figure 4d) (Figure 4c, 47.73 ± 5.14 vs. 28.20 ± 1.18, *p*< 0.0001, *n* = 4) and caspase‐1(pro) (Figure 4a) (Figure 4d) (Figure 4f, 1.08 ± 0.14 vs. 0.35 ± 0.06, *p*< 0.0001, *n* = 3), caspase‐1(cleavad) (Figure 4a) (Figure 4d) (Figure 4f, 1.13 ± 0.06 vs. 0.33 ± 0.06, *p*< 0.0001, *n* = 3) was elevated. However, P‐mTOR (Figure 4a) (Figure 4d) (Figure 4g, 1.07 ± 0.15 vs. 1.75 ± 0.16, *p*< 0.0001, *n* = 3), NLRP3 (Figure 4b) (Figure 4d) (Figure 4c, 34.94 ± 1.63 vs. 47.73 ± 5.14, *p*< 0.0001, *n* = 4), and caspase‐1(pro) (Figure 4a) (Figure 4d) (Figure 4f, 0.66 ± 0.06 vs. 1.08 ± 0.14, *p*<0.01, *n* = 3), and caspase‐1(cleavad) (Figure 4a) (Figure 4d) (Figure 4f, 0.60 ± 0.04 vs. 1.13 ± 0.06, *p*< 0.0001, *n* = 3) expression was lower in the “rapa‐lipos” group than in the “IL‐1*β*” group. These data suggest that IL‐1*β* induces senescence in NP cells, leading to increased P‐mOR levels, which in turn leads to increased expression of NLRP3 and caspase‐1 in the NCP. After combined treatment of NP cells with rapa‐lipos+IL‐1*β*, P‐mOR levels and NCP‐related proteins also decreased. Therefore, rapa‐lipos can inhibit the NCP in an inflammatory environment.

For the ALP‐related proteins, LC3II/LC3I ratio (Figure 4a) (Figure 4d) (Figure 4h, 0.43 ± 0.04 vs. 0.79 ± 0.07, *p*< 0.01, *n* = 3) expression was decreased, and P62 (Figure 4a) (Figure 4d) (Figure 4i, 1.36 ± 0.29 vs. 0.48 ± 0.04, *p*< 0.0001, *n* = 3) expression was increased when G0 NP cells were stimulated with IL‐1*β*. Moreover, the expression of LC3II/LC3I ratio (Figure 4a) (Figure 4d) (Figure 4h, 0.43 ± 0.04 vs. 0.62 ± 0.07, *p*< 0.05, *n* = 3) was greater and that of P62 (Figure 4a) (Figure 4d) (Figure 4i, 0.78 ± 0.11 vs. 1.36 ± 0.29, *p*< 0.01, *n* = 3) was lower in the “rapa‐lipos” group than in the “IL‐1*β*” group. These results indicating that rapa‐lipos can promote autophagy–lysosomal function in ALP.

PCR: The PCR results revealed that after IL‐1*β* stimulation of G0 NP cells, the expression of the IL‐1R gene (Figure 4d) (Figure 4j, 1.76 ± 0.09 vs. 1.01 ± 0.21, *p*< 0.01, *n* = 3), the IL‐1*β* gene (Figure 4d) (Figure 4k, 2.44 ± 0.14 vs. 1.01 ± 0.14 *p*< 0.0001, *n* = 3), and the P62 gene (Figure 4d) (Figure 4m, 3.92 ± 0.10 vs. 1.02 ± 0.22, *p*< 0.0001, *n* = 3) increased, whereas the expression of the LC3 gene (Figure 4d) (Figure 4l, 0.17 ± 0.02 vs. 1.01 ± 0.14, *p*< 0.0001, *n* = 3) decreased. Compared with those in the “IL‐1*β*” treatment group, the levels of the IL‐1*β* gene (Figure 4d) (Figure 4k, 1.43 ± 0.19 vs. 2.44 ± 0.14, *p*< 0.0001, *n* = 3) and P62 gene (Figure 4d) (Figure 4m, 0.79 ± 0.05 vs. 3.92 ± 0.10, *p*< 0.0001, *n* = 3) were decreased, whereas the LC3 gene level (Figure 4d) (Figure 4l, 0.96 ± 0.05 vs. 0.17 ± 0.02, *p*< 0.0001, *n* = 3) was increased, and the expression of the IL‐1R gene (Figure 4d) (Figure 4j, 1.78 ± 0.28 vs. 1.76 ± 0.09, *p* >0.05, *n* = 3) was unchanged in the “rapa‐lipo” group. The gene expression of IL‐1R (Figure 4d) (Figure 4j, *p* >0.05, *n* = 3), IL‐1*β* (Figure 4d) (Figure 4k, *p* >0.05, *n* = 3), P62 (Figure 4d) (Figure 4m, *p* >0.05, *n* = 3), and LC3 (Figure 4d) (Figure 4l, *p* >0.05, *n* = 3) was not significantly different or physiologically significant between the “Blank” and “G0” groups. Therefore, the PCR results revealed that the expression levels of genes related to the ALP and NCP were directly proportional to protein expression in the WB.

### 3.5 The mechanism by which rapa‐lipos target mTORC1 was further verified: siRNA‐raptors block mTORC1 (WB, IF)

The experiments in the previous section confirmed that the rapa‐lipos inhibits mTORC1 phosphorylation, leading to the inhibition of the NCP and the activation of the ALP. To further verify the molecular effect of inhibiting mTORC1, we silenced mTORC1 via siRNA technology and further analyzed the changes in mechanism in the NCP and ALP.

WB and IF results revealed that after G0 NP cells were stimulated with IL‐1*β*, the expression of IL‐1R increased (Figure 5a) (Figure 5d) (Figure 5e, 0.76 ± 0.10 vs. 0.45 ± 0.10, *p*< 0.01; *n* = 3). Compared with IL‐1*β* stimulation, stimulation with IL‐1*β* + Si‐RNA (raptor) did not significantly affect IL‐1R expression (Figure 5a) (Figure 5d) (Figure 5e, 0.76 ± 0.10 vs. 0.80 ± 0.01, *p* >0.05; *n* = 3).

**FIGURE 5:**
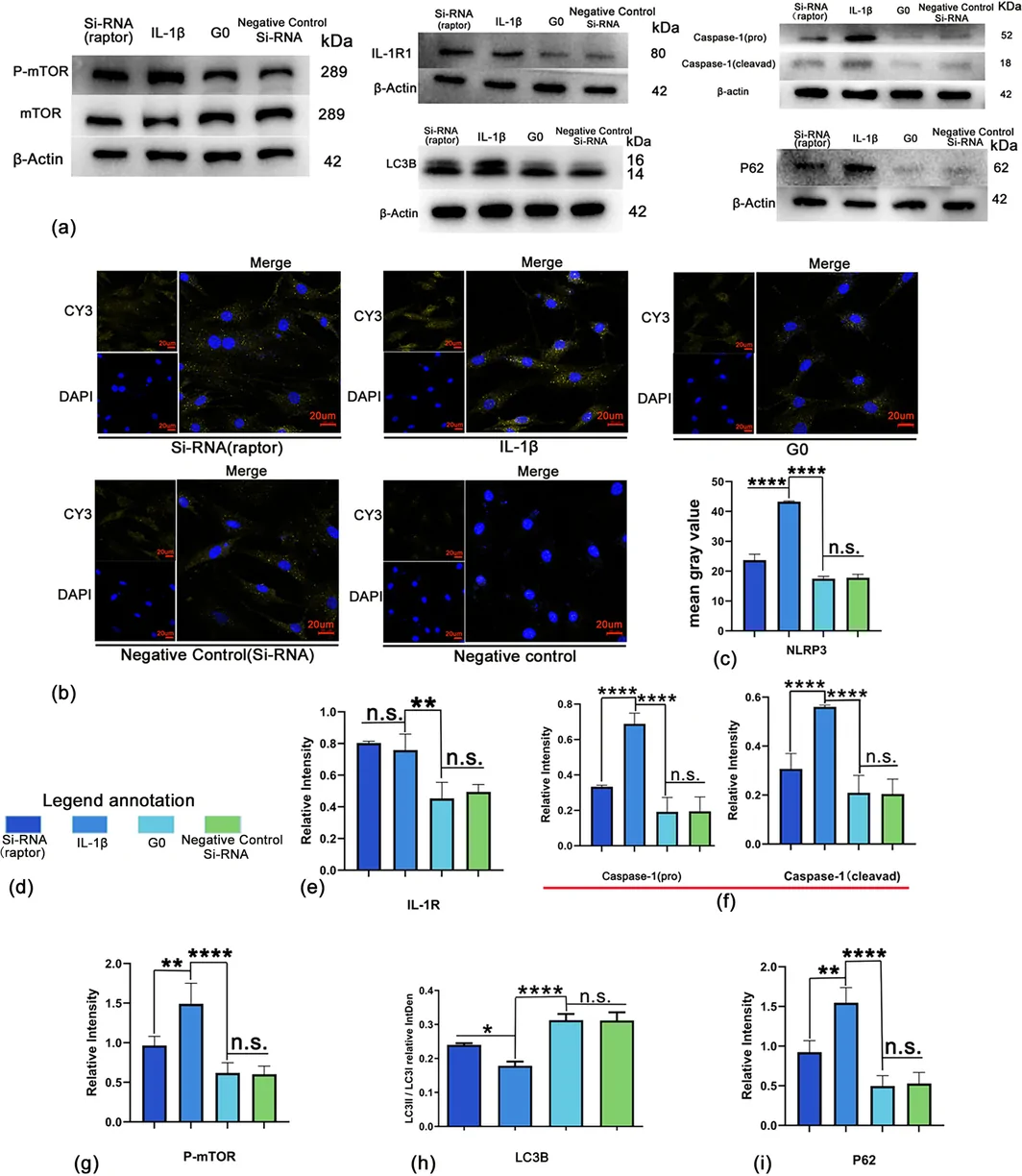
Effects of siRNA (raptor) mediated silencing of mTORC1 on the molecular mechanism of NCP and ALP. NP cells were subjected to WB (a) and IF (b) for P‐mTOR (g), the NCP‐associated proteins IL‐1R (e), NLRP3 (c), and Caspase‐1(pro) and Caspase‐1(cleavad) (f); and the ALP‐associated proteins LC3 (h) and P62 (i). (WB: *N* = 3, IF: *N* = 4; mean ± SD, one‐way ANOVA, *p*< 0.05 indicates a statistically significant difference, *p< 0.05, **p< 0.01, ****p< 0.0001, n.s. indicates no significance.) The legend annotation (d).

After IL‐1*β* stimulation of G0 NP cells, P‐mTOR (Figure 5a) (Figure 5d) (Figure 5g, 1.49 ± 0.26 vs. 0.62 ± 0.13, *p*< 0.0001, *n* = 3), NCP‐associated protein NLRP3 (Figure 5b) (Figure 5d) (Figure 5c, 43.25 ± 0.28 vs. 17.47 ± 0.79, *p*< 0.0001, *n* = 4), caspase‐1(pro) (Figure 5a) (Figure 5d) (Figure 5f, 0.69 ± 0.06 vs. 0.19 ± 0.08, *p*< 0.0001, *n* = 3) and caspase‐1(cleavad) (Figure 5a) (Figure 5d) (Figure 5f, 0.56 ± 0.01 vs. 0.21 ± 0.07, *p*< 0.0001, *n* = 3) expression was also elevated. Compared with those after IL‐1*β* stimulation, the expression of P‐mTOR (Figure 5a) (Figure 5d) (Figure 5g, 0.97 ± 0.11 vs. 1.49 ± 0.26, *p*< 0.01, *n* = 3), NLRP3 (Figure 5b) (Figure 5d) (Figure 5c, 23.67 ± 2.00 vs. 43.25 ± 0.28, *p*< 0.0001, *n* = 4), and caspase‐1(pro) (Figure 5a) (Figure 5d) (Figure 5f, 0.33 ± 0.01 vs. 0.69 ± 0.06, *p*< 0.0001, *n* = 3) and caspase‐1(cleavad) (Figure 5a) (Figure 5d) (Figure 5f, 0.30 ± 0.06 vs. 0.56 ± 0.01, *p*< 0.0001, *n* = 3) was decreased in the “Si‐RNA (raptor)” group.

For the ALP‐associated proteins, G0 NP cells stimulated with IL‐1*β* presented increased expression of P62 (Figure 5a) (Figure 5d) (Figure 5i, 1.55 ± 0.19 vs. 0.50 ± 0.13, *p*< 0.0001, *n* = 3). Compared with that after IL‐1*β* stimulation, after stimulation with IL‐1*β* + Si‐RNA (raptor), P62 expression decreased (Figure 5a) (Figure 5d) (Figure 5i, 0.92 ± 0.14 vs. 1.55 ± 0.19, *p*< 0.01, *n* = 3). To further detect the effect of siRNA (raptor) on autophagy flow, when NP cells treated with IL‐1*β*, the LC3II/LC3I ratio was decreased compared with that in the G0 group (Figure 5a) (Figure 5d) (Figure 5h, 0.18 ± 0.02 vs. 0.31 ± 0.03, *p*< 0.0001, *n* = 3). NP cells transfected with siRNA (raptor) were treated with IL‐1*β*, and the LC3II/LC3I ratio was increased compared with that in the IL‐1*β* group (Figure 5a) (Figure 5d) (Figure 5h, 0.24 ± 0.09 vs. 0.18 ± 0.02, *p*< 0.05, n = 3), indicating that Si‐RNA (raptor) transfected NP cells can promote autophagic flow.

The expression levels of NCP‐related proteins and ALP‐related proteins in NP cells stimulated with negative control siRNA did not significantly differ from those in G0 NP cells.

In summary, by silencing mTORC1 with a SiRNA (raptor), we observed a molecular effect similar to that of the inhibition of mTORC1 by rapa‐lipos. Compared with IL‐1*β* treatment, IL‐1*β* + Si‐RNA(raptor) treatment of NP cells inhibited the NCP and enhanced the ALP at the protein levels. Therefore, we conclude that rapa‐lipos targets mTORC1 and then mediates the NCP and ALP to inhibit NP cell senescence.

### 3.6 Rapa‐lipos regulates cell cycle inhibitors to maintain NP cell quiescence

Cellular senescence is characterized by permanent cell cycle arrest, accompanied by elevated expression of P16 and P21; in contrast, cellular quiescence features reversible cell cycle arrest, typically marked by upregulated P27 expression.^28^ We further evaluated the effects of rapa‐lipos on cellular senescence and quiescence by detecting the expression levels of P16, P21, and P27.

Treatment with rapa‐lipos induced alterations in the expression of these cell cycle‐related proteins across groups. Compared with the G0 group, the IL‐1*β* group exhibited significantly increased P16 expression (Figure 6a,b,f; 1.13 ± 0.09 vs. 0.41 ± 0.09, *p*< 0.0001, *n* = 3) and P21 expression (Figure 6d–f; 1.05 ± 0.08 vs. 0.46 ± 0.09, *p*< 0.0001, *n* = 3), but markedly decreased P27 expression (Figure 6a,c,f; 0.44 ± 0.07 vs. 0.91 ± 0.17, *p*< 0.01, *n* = 3). In comparison with the IL‐1*β* group, the rapa‐lipos group showed reduced expression of both P16 (Figure 6a,b,f; 0.67 ± 0.13 vs. 1.13 ± 0.09, *p*< 0.0001, *n* = 3) and P21 (Figure 6d–f; 0.71 ± 0.06 vs. 1.05 ± 0.08, *p*< 0.01, *n* = 3), whereas P27 expression was significantly upregulated (Figure 6a,c,f; 0.86 ± 0.13 vs. 0.44 ± 0.07, *p*< 0.01, *n* = 3). No significant differences in P16, P21, and P27 expression were observed between the G0 group and the blank‐lipos group.

**FIGURE 6:**
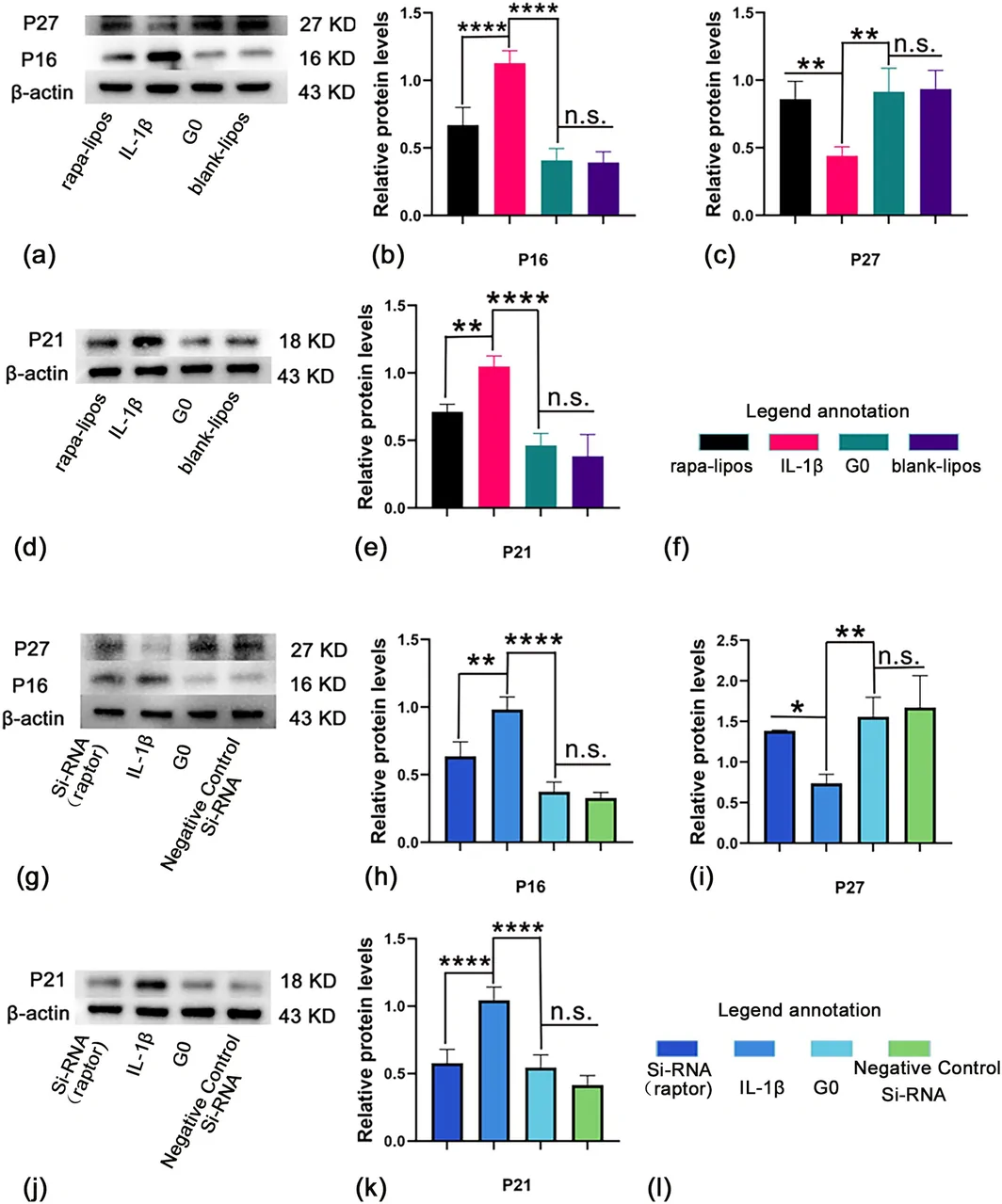
Rapa‐lipos downregulates P16/P21 and upregulates P27 to maintain NP cell quiescence. (a) Representative WB of P16 and P27 in rapa‐lipos treated NP cells (groups: G0, IL‐1*β*, rapa‐lipos, blank‐lipos). (b), (c) Quantitative analysis of P16 (b) and P27 (c) in (a). (d) Representative WB of P21 (same groups as a). (e) Quantitative analysis of P21 in (d). (f) Group legend for (a)–(e) (rapa‐lipos treated). (g) Representative WB of P16 and P27 in NP cells transfected with Si‐RNA(raptor) (groups: G0, IL‐1*β*, Si‐RNA(raptor), Negative control Si‐RNA). (h), (i) Quantitative analysis of P16 (h) and P27 (i) in (g). (j) Representative WB of P21 (same groups as g). (k) Quantitative analysis of P21 in (j). (l) Group legend for (g)–(k) (Si‐RNA(raptor) transfected). (*n* = 3, mean ± SD, one‐way ANOVA, *p*< 0.05 indicates a statistically significant difference, *p< 0.05, **p< 0.01, ****p< 0.0001).

NP cells transfected with Si‐RNA(raptor) displayed similar trends in the expression of these cell cycle inhibitors as the rapa‐lipos treated group. Relative to the G0 group, the IL‐1*β* group had higher P16 expression (Figure 6g,h,l; 0.98 ± 0.09 vs. 0.37 ± 0.07, *p*< 0.0001, *n* = 3) and P21 expression (Figure 6j–l; 1.04 ± 0.10 vs. 0.54 ± 0.09, *p*< 0.0001, *n* = 3), but lower P27 expression (Figure 6g,i,l; 0.74 ± 0.11 vs. 1.56 ± 0.24, *p*< 0.01, *n* = 3). When compared with the IL‐1*β* group, the Si‐RNA(raptor) group presented decreased P16 (Figure 6g,h,l; 0.64 ± 0.11 vs. 0.98 ± 0.09, *p*< 0.01, *n* = 3) and P21 expression (Figure 6j–l; 0.58 ± 0.10 vs. 1.04 ± 0.10, *p*< 0.0001, *n* = 3), along with increased P27 expression (Figure 6g,i,l; 1.38 ± 0.01 vs. 0.74 ± 0.11, *p*< 0.05, *n* = 3). Additionally, there were no notable differences in the expression of P16, P21, and P27 between the G0 group and the negative control Si‐RNA group.

In conclusion, under low‐nutrient conditions, NP cell senescence is associated with upregulated P16 and P21 expression and downregulated P27 expression, and these changes can be reversed by rapa‐lipos treatment. Collectively, these findings indicate that rapa‐lipos can maintain NP cell quiescence and inhibit cellular senescence.

### 3.7 Study of rapa‐lipos delaying the degeneration of rat caudal intervertebral discs

Disc degeneration is characterized by changed morphology, reduced disc space, increased Pfirrmann score, and attenuated fluorescence intensity of aggrecan.^30^ This section focuses on the mechanism by which rapa‐lipos inhibits the degeneration of the intervertebral disc.

X‐ray imaging: Rat (BEIJING HFK BIOSCIENCE CO., Ltd., 8 weeks old, 180–220 g, male) caudal intervertebral discs injected with IL‐1*β*, IL‐1*β* + rapa‐lipos, or blank liposomes for 1 month presented different degrees of reduction in disc height than normal discs did (Figure 7b). Compared with untreated, IL‐1*β* decreased the height most significantly (Figure 7f) (Figure 7d, 40.83 ± 17.71 vs. 100, *n* = 4, *p*< 0.0001). Compared with that of the discs injected with IL‐1*β* alone, the DHI of the discs treated with IL‐1*β* + rapa‐lipos was lower (Figure 7f) (Figure 7d, 75 ± 10 vs. 40.83 ± 17.71, *n* = 4, *p*< 0.0001).

**FIGURE 7:**
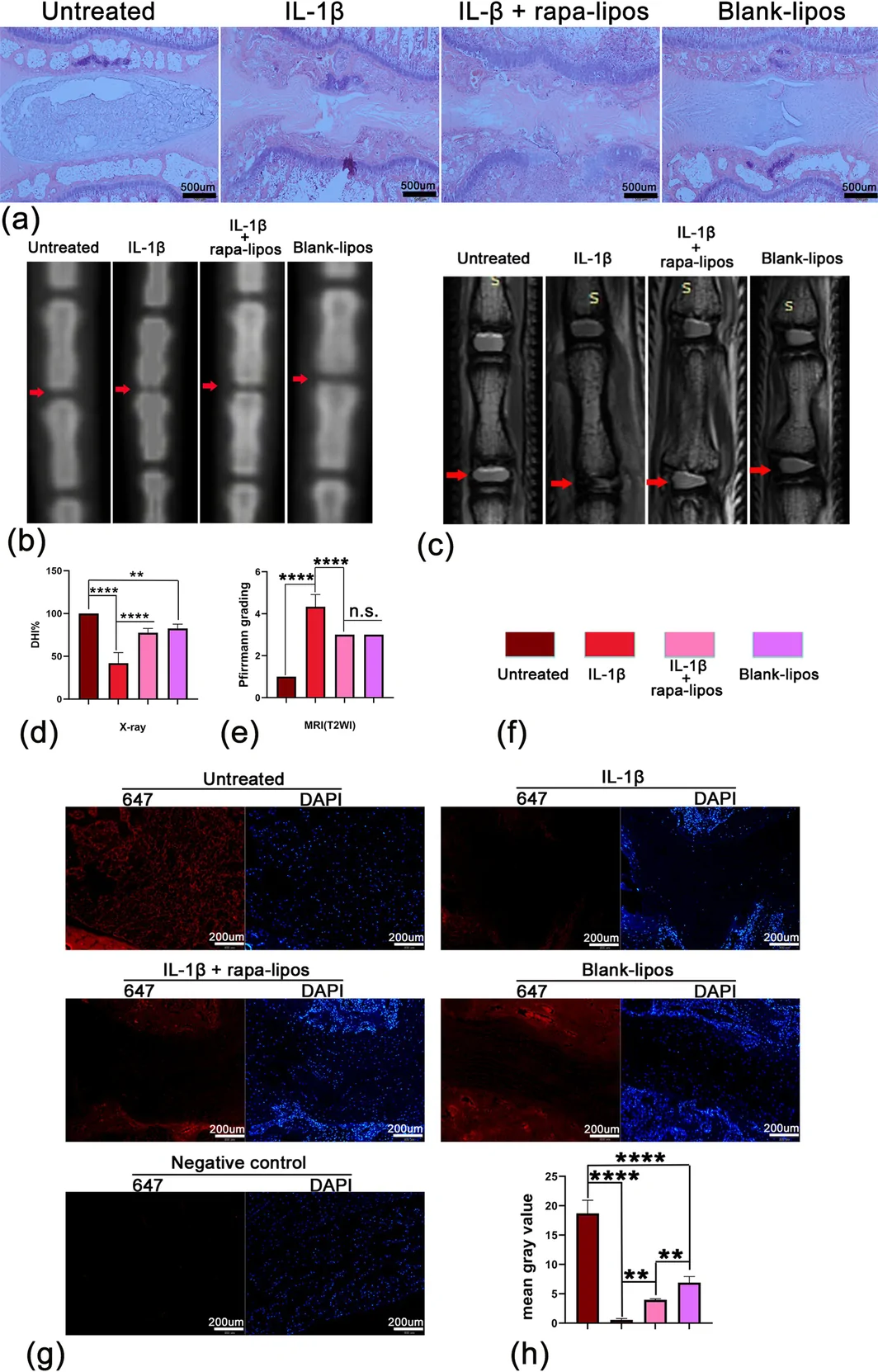
Degeneration of intervertebral discs in the tails of rats subjected to different treatments. HE staining (a) was used to evaluate the gross morphology of degenerated intervertebral disc. X‐ray imaging (b) was used to evaluate the changes in the vertebral space. MRI observations (T2W1) (c) for rat caudal intercalated discs. The disc height index (d) (disc height index, DHI) were used to analyze x‐ray for the degeneration of the intervertebral discs (*n* = 4, mean ± SD, one‐way ANOVA, *p*< 0.05 indicates a statistically significant difference, *p< 0.05, **p< 0.01, ****p< 0.0001, n.s. indicates no significance). The Pfirrmann scoring system (e) were analyzed MRI images for the degeneration of rat caudal intercalated discs. (*n* = 3, mean ± SD, one‐way ANOVA; *p*< 0.05 indicates a statistically significant difference, **p< 0.01, ****p< 0.0001, n.s. indicates no significance.) The legend annotation (f). Immunofluorescence staining (g) and correlation fluorescence intensity analysis (h) of extracellular matrix component aggrecan (*n* = 3, mean ± SD, one‐way ANOVA, *p*< 0.05 indicates a statistically significant difference, **p< 0.01, ****p< 0.0001).

*MRI*: One month after the injection of different drugs (such as “IL‐1*β*,” “IL‐1*β* + rapa‐lipos,” or “blank liposomes”) into rat caudal intervertebral discs, MR images were taken and scored via the “Pfirrmann method.” Treatment with IL‐1*β*, IL‐1*β* + rapa‐lipos, or blank liposomes resulted in different degrees of disc degeneration (Figure 7c). For Pfirrmann score, the “IL‐1*β*” group is (4.33 ± 0.58), the “IL‐1*β* + rapa‐lipos” (3.00 ± 0.00) and “blank liposome” groups (3.00 ± 0.00) (Figure 7f) (Figure 7e). The degeneration score of the “IL‐1*β* + rapa‐lipos” group is significantly lower than that of the “IL‐1*β*” group (Figure 7f) (Figure 7e, *n* = 3, *p*< 0.0001). There was no statistically significant difference in the degeneration scores of the “blank liposomes” and “IL‐1*β* + rapa‐lipos” groups (Figure 7f) (Figure 7e, *n* = 3, *p* >0.05).

*HE staining*: In the untreated group, there was internal nucleus pulposus tissue and no collapse of intervertebral disc (Figure 7a). When the disc was injected into blank liposomes, there was slight space collapse (Figure 7a). After IL‐1*β* treatment, the nucleus pulposus tissue disappeared and the intervertebral space was obviously narrow (Figure 7a). However, after IL‐*1β* + rapa‐lipos treatment, there was a certain space in the intervertebral space, and the tissue characteristics of the nucleus pulposus changed (Figure 7a).

IF staining: After anti‐aggrecan fluorescence staining, nucleus pulposus tissues showed different fluorescence intensities (Figure 7g). Compared to the “untreated group,” the “IL‐1*β*” group showed decreased mean fluorescence intensity (Figure 7f) (Figure 7h, 0.522 ± 0.23048 vs. 18.6733 ± 2.24475, *n* = 3, *p*< 0.0001), and the “blank liposomes” group also showed decreased (Figure 7f) (Figure 7h, 6.8907 ± 1.041 vs. 18.6733 ± 2.24475, *n* = 3, *p*< 0.0001). Compared with the “IL‐1*β*” group, the mean fluorescence intensity of “IL‐1*β* + rapa‐lipos” group was enhanced (Figure 7f) (Figure 7h, 3.9513 ± 0.17981 vs. 0.522 ± 0.23048, *n* = 3, *p*< 0.01). Compared with the “IL‐1*β* + rapa‐lipos” group, “blank liposomes” group also showed increases in the mean fluorescence intensity (Figure 7f) (Figure 7h, 6.8907 ± 1.041 vs. 3.9513 ± 0.17981, *n* = 3, *p*< 0.01).

## 4 DISCUSSION

In this study, NP cell senescence is characterized by growth arrest, changes in cell morphology, increased *β*‐gal, increased inflammatory secretion, altered expression of cell cycle inhibitors, and lysosome dysfunction. However, rapa‐lipos can inhibit the above‐described changes. Molecular mechanism analysis revealed that, compared with senescent NP cells, rapa‐lipos + IL‐1*β* treatment reduced P‐mTORC1 levels, inhibited NCP activation, activated the ALP, and maintained cell quiescence to resist cellular senescence. In addition, we found that intervertebral disc degeneration was associated with changed morphology, intervertebral space narrowing, and increased Pfirrmann score. Rapa‐lipos also restrained these changes in intervertebral disc degeneration. Therefore, rapa‐lipos not only inhibits cell senescence but also disc degeneration.

In the first step of this experiment, we used a simple preparation process to construct rapa‐lipos with high drug loading rates and encapsulation rates, long sustained‐release times of rapamycin, and uniform particle sizes. A key factor in liposome production is encapsulation efficiency (EE%).^31^ We determined that the EE% of the rapa‐lipos was 80.36% ± 3.99% and that the liposomes were able to controlled‐release rapamycin for at least 28 days. The EE% of rapa‐lipos prepared by Miao et al.^32^ was 82.11% ± 2.13%. Additionally, the EE% of rapa‐lipos prepared by Chen et al.^33^ was 80% ± 2.5%. The experimental EE% in our study was relatively high, which may be due to the proper ratio of phospholipids, alginate, and rapamycin. Previous studies have shown that^34^ the addition of alginose during liposome fabrication can be used to protect the lipid system from chemical and physical changes during freezing and thawing and to maintain the drug loading capacity of liposomes. The addition of alginose to the liposome construct used in this study might have helped increase the EE%. Thus, rapa‐lipos constructed from phospholipids, alginose, and rapamycin are soluble in water and have a high encapsulation rate and the ability to release drugs in a controlled manner. In the preparation process, the suspension of the rapa‐lipos was filtered through a 0.45 μm filter, which is the key point for obtaining a uniform particle size.^35^ Therefore, in this study, ultrasonic dispersion, thin film dispersion and filtering methods were adopted, and the prepared rapa‐lipos exhibited excellent performance and may be a better carrier for studying cellular senescence.

Cellular senescence is a stable, growth‐arrested end state.^26^, ^36^, ^37^ However, cell quiescence is not only a cell cycle without proliferation, but also a young state of the cell.^4^ Rapamycin, an inhibitor of mTORC1, effectively inhibits cellular senescence.^38^ Due to its high loading rate with rapamycin, rapa‐lipos is highly efficient for inhibiting cell senescence.^39^ Growth arrest in the context of cellular senescence manifests as inactivation of the cell cycle and increased expression of the cellular senescence marker *β*‐gal.^40^, ^41^ Similar conclusions were obtained in this study with *β*‐gal, EdU staining, and flow cytometry: NP cells cultured under low‐nutrient conditions were arrested in the G0/G1 phase and maintained cell quiescence. In contrast, senescent NP cells not only had growth arrest, but also increased *β*‐gal expression. However, the rapa‐lipos inhibited the expression of *β*‐gal and maintained cell quiescence. In a previous experimental study, Li et al.^3^ reported that low‐nutrient conditions or rapamycin can maintain the cell quiescence. The difference in this study is how rapa‐lipos efficiently maintain the quiescence of NP cells in the inflammatory microenvironment, and the molecular mechanisms associated with autophagy–lysosome dysregulation and inflammatory secretion were also analyzed.

Cellular senescence is characterized by irreversible cell cycle arrest, a phenotype measurable via cell cycle inhibitors. In the present study, rapa‐lipos suppressed NP cell senescence, as evidenced by decreased P21 or P16 and increased P27 expression. A shared hallmark of cellular senescence and quiescence is cell cycle arrest.^42^ Specifically, cellular senescence is defined as irreversible cell cycle arrest that occurs independently of mTOR inhibition, accompanied by substantial accumulation of the P21 and the P16.^43^, ^44^ By contrast, cell cycle arrest associated with mTOR inhibition is termed cellular quiescence, which can induce lysosomal autophagy via the P27.^8^, ^45^ Li et al.^3^ demonstrated that NP cells enter a quiescent state under nutrient deprivation conditions, as evidenced by increased P27 expression. Fan et al.^46^ further confirmed that high‐density cell culture triggers contact inhibition, driving cells into quiescence with a concomitant upregulation of P27. Collectively, these findings indicate that increased P27 expression is indicative of enhanced anti‐senescence capacity in cells. Our findings echoed these observations: rapa‐lipos preserved NP cell quiescence and suppressed senescence, as reflected by reduced P21/P16 and elevated P27 expression.

Another important feature of senescent cells is lysosomal dysfunction.^47^ In the present study, TEM and fluorescent lysosomal tracer staining revealed that lysosomal structure was disrupted and the number of lysosomes was increased in senescent NP cells, while rapa‐lipos blocked these changes in lysosomes. This change was due to the dysfunction of lysosomes in senescent cells and the addition number of lysosomes enhances cellular lysosomal function.^26^, ^48^ Using TEM in conjunction with lysosomal tracer staining, Tai et al.^26^ reached the same conclusions, with an increase in the number of dysfunctional lysosomes in senescent cells. García‐Prat et al.^48^ reported that the number of dysfunctional lysosomal vesicles was increased in senescent tibialis anterior muscle satellite cells, whereas the number was relatively reduced in young cells. Lawrence et al.^49^ reported that lysosomes are extremely important in the maintenance of tissue and cellular health and the prevention of aging‐associated senescence. Therefore, in the present study, an important hallmark of NP cell senescence was autophagy–lysosome dysfunction, which can be prevented by the use of rapa‐lipos.

Senescent cells also exhibit increased secretion of senescence‐associated secretory phenotype (SASP) proteins, which include proinflammatory and immunomodulatory cytokines and chemokines.^36^ IL‐1*β* is a core cytokine of the SASP and has been shown to be involved in the pathogenesis of intervertebral disc degeneration.^50^, ^51^ In our present study, ELISA revealed that senescent NP cells increased the secretion of IL‐1*β* and TNF‐*α*, whereas rapa‐lipos inhibited this secretion.

The core mechanism by which rapa‐lipos inhibit NP senescence is the inhibition of mTORC1 phosphorylation, which leads to an increase in lysosomal autophagy.^12^ In this study, we showed that rapa‐lipos inhibited mTORC1 and increased NP cell autophagy, leading to a decrease in the levels of the associated protein P62 (SQSTM1 protein) and an increase in the ratio of LC3II/LC3I, and maintenance of the quiescent (G0 phase) phase in NP cells. Lu et al.^52^ verified that increased autophagy in decidual stromal cells prevented spontaneous abortion, and the mechanism involved a decrease in P62 levels and an increase in the LC3II/I ratio. Joseph et al.^9^ also reported that inhibiting mTORC1 led to an increased autophagy flow (LC3II/I) in skeletal muscle and delayed the progression of skeletal sarcopenia. Moreover, Altman et al.^53^ reported that autophagy was activated after rapamycin inhibited mTORC1.

Another core mechanism by which rapa‐lipos inhibit NP cell senescence is the inhibition of translation downstream of mTORC1 and then the inhibition of the NCP, which can reduce SASP secretion (such as IL‐1*β*, TNF‐*α*). Li et al.^29^ demonstrated that rapamycin attenuates sepsis‐induced damage to tissues through autophagy‐mediated inactivation of the NLRP3 inflammasome. Majumder et al.^54^ demonstrated that prolonged use of rapamycin reduced IL‐1*β* secretion in the brain and alleviated aging‐induced cognitive deficits. Lackner et al.^55^ confirmed that caspase‐1 (cleaved) represents the activity of the NLRP3 inflammasome, and therefore the NLRP3 pyrin domain inhibitors directly target and inhibit NLRP3, preventing the activation of the inflammasome and the production of IL‐1*β*. Limbad et al.^56^ suggested that rapamycin was able to inhibit the SASP by inhibiting IL‐1a translation through the suppression of the mTOR pathway.

To establish an intervertebral disc degeneration model, IL‐1*β* was microinjected into the rat caudal intervertebral disc to induce degeneration. However, this degeneration was delayed by coinjection of IL‐1*β* + rapa‐lipos. Kim et al.^57^ also demonstrated that the injection of IL‐1*β* can be used to successfully establish a model of intervertebral disc degeneration. Chen et al.^58^ used a 21‐gauge needle to inject IL‐1*β* into the intervertebral disc to induce disc degeneration; the needle was inserted 3.0 mm into the tissue and left in the skin for 30 s. In contrast, the microsyringe used in our study to puncture the disc transiently resulted in slight degeneration of the disc after 1 month, probably because we used a thinner needle and inserted it into the disc more superficially and for a shorter duration. Many scholars have used hydrogels to treat intervertebral disc degeneration. However, owing to the limited volume of drugs that reach the intervertebral disc, 2–10 μL of the hydrogels are generally injected.^59^, ^60^, ^61^ We chose to construct rapa‐lipos because it can be used in a relatively small volume, which can increase the ability of rapamycin to enter the intervertebral disc, subsequently delay intervertebral disc degeneration.

The rapa‐lipos inhibits NP cell senescence and disc degeneration because NP cells are in a sealed microenvironment inside the disc and lack blood flow to supply nutrients, resulting in NP cell quiescence.^1^, ^2^ In our research, rapa‐lipos can regulate the ALP and NCP by controlling mTORC1 phosphorylation levels to achieve NP cell quiescence. However, rapa‐lipos are not necessarily suitable for regulating the aging of other cells because the regulation of mTORC1 may lead to the restriction of cell metabolism, growth, and substance synthesis.^62^ This is the flaw in the study of rapa‐lipos. In later studies, we will investigate how rapa‐lipos in vivo affects the molecular mechanisms of mTORC1, ALP, and NCP. It is more beneficial to confirm the molecular mechanism of rapa‐lipos inhibiting intervertebral disc degeneration. At the same time, we plan to conduct long‐term safety experiments of rapa‐lipos in SD rats, such as continuous administration for 6, 12 months, and so forth,^63^ to provide data support for the clinical application of intervertebral disc inhibition.

## 5 CONCLUSION

Our data suggest that rapa‐lipos that are prepared via ultrasonic dispersion, thin film dispersion and the filter method have the following advantages: a high drug loading rate and encapsulation rate, a long sustained release time of rapamycin, and a uniform particle size. Therefore, it is beneficial to the research of cell senescence. When mTORC1 phosphorylation is increased in senescent NP cells, lysosomal autophagy is impaired, and inflammatory factors (such as IL‐1*β*, TNF‐*α*) are secreted. The rapa‐lipos targeting mTORC1 were able to activate the ALP while inhibiting the NCP and thereby inhibiting inflammatory secretion and maintaining the NP cells' quiescence. The rapa‐lipos also prevented disc degeneration in vivo. In conclusion, rapa‐lipos have the advantages of simple preparation and excellent performance, which can block the senescence of NP cells and intervertebral disc degeneration. Therefore, the use of rapa‐lipos may provide new hope for the treatment of intervertebral disc degeneration in humans in the future.

## 6 CLINICAL PERSPECTIVE

Point1: NP cells reside in the avascular, enclosed microenvironment of intervertebral discs, with limited nutrient supply that maintains their quiescent state and intervertebral discs homeostasis. During intervertebral discs degeneration, inflammatory infiltration induces NP cell senescence, exacerbating degenerative progression. Rapamycin sustains NP cell quiescence via targeted inhibition of mTORC1 phosphorylation, suggesting its potential to preserve NP cell quiescence and delay intervertebral discs degeneration under inflammatory conditions.

Point2: To improve rapamycin's water solubility, we fabricated rapamycin‐liposomes (rapa‐lipos)—ideal carriers with high encapsulation efficiency, favorable drug loading, uniform particle size, and controlled release—facilitating investigations into cellular senescence. NP cell senescence is characterized by morphological alterations, elevated secretion of inflammatory SASP factors (e.g., IL‐1*β*, TNF‐*α*), increased *β*‐gal activity, and lysosomal dysfunction, all of which are attenuated by rapa‐lipos. Mechanistically, rapa‐lipos suppress mTORC1 phosphorylation, leading to NCP inactivation, ALP activation, and preservation of NP cell quiescence, thereby abrogating cellular senescence. In vivo evaluations via x‐ray, MRI, and histology further confirmed that rapa‐lipos prevent intervertebral discs degeneration.

Point3: Collectively, rapa‐lipos successfully target mTORC1 to inhibit NP cell senescence, exerting a protective effect against intervertebral disc degeneration.

## AUTHOR CONTRIBUTIONS

**Hui Xing:** Methodology; visualization; formal analysis; writing – original draft; writing – review and editing. **Quanfang Wei:** Resources. **Yu Guo:** Resources. **Miao Yu:** Investigation; data curation. **Xuezheng Ai:** Investigation. **Runzhi Zhao:** Investigation. **Tong Zhu:** Investigation. **Lu Jiang:** Investigation. **Youying Huang:** Resources. **Tao Jiang:** Conceptualization; methodology; funding acquisition; writing – review and editing; supervision. **Yuqing Li:** Investigation. **Rong Tang:** Investigation. **Jiabin Liu:** Data curation; investigation. **Bo Huang:** Conceptualization; methodology; funding acquisition; writing – review and editing; supervision.

## FUNDING INFORMATION

This research was supported by the National Natural Science Foundation of China (Grant No: 8197091181) and Natural Science Foundation of Chongqing (Grant No. cstc2020jcyj‐msxmX0278).

## CONFLICT OF INTEREST STATEMENT

The authors have no financial conflict of interest to declare.

## Data Availability

The data that support the findings of this study are available from the corresponding author upon reasonable request.
